# Impact of Perioperative Dexamethasone Administration on Infection and Implant Osseointegration in a Preclinical Model of Orthopedic Device-Related Infection

**DOI:** 10.3390/microorganisms12061134

**Published:** 2024-06-01

**Authors:** Marc-Antoine Burch, Aron Keshishian, Charlotte Wittmann, Dirk Nehrbass, Keith Thompson, Daniel Arens, R. Geoff Richards, Vuysa Mdingi, Marco Chitto, Mario Morgenstern, T. Fintan Moriarty, Henk Eijer

**Affiliations:** 1 AO Research Institute Davos, 7270 Davos, Switzerland; marc-antoine.burch@usb.ch (M.-A.B.);; 2 Klinik für Orthopädie und Traumatologie, Universitätsspital Basel, 4031 Basel, Switzerland; 3 Spital Emmental, 3400 Burgdorf, Switzerland; 4 Department of Orthopaedic Surgery, Dr Pixley Ka Isaka Seme Memorial Hospital, School of Clinical Medicine, University of KwaZulu Natal, Durban 4041, South Africa; 5 Center for Muskuloskeletal Infections (ZMSI), University Hospital Basel, 4031 Basel, Switzerland

**Keywords:** *Staphylococcus epidermidis*, periprosthetic joint infection, PJI, fracture-related infection, FRI, orthopedic-device-related infection, ODRI, dexamethasone, osteomyelitis, in vivo microCT, outpatient total joint replacement

## Abstract

Glucocorticoids may be given prior to major orthopedic surgery to decrease postoperative nausea, vomiting, and pain. Additionally, many orthopedic patients may be on chronic glucocorticoid therapy. The aim of our study was to investigate whether glucocorticoid administration influences Orthopedic-Device-Related Infection (ODRI) in a rat model. Screws colonized with *Staphylococcus epidermidis* were implanted in the tibia of skeletally mature female Wistar rats. The treated groups received either a single shot of dexamethasone in a short-term risk study, or a daily dose of dexamethasone in a longer-term interference study. In both phases, bone changes in the vicinity of the implant were monitored with microCT. There were no statistically significant differences in bacteriological outcome with or without dexamethasone. In the interference study, new bone formation was statistically higher in the dexamethasone-treated group (*p* = 0.0005) as revealed by CT and histopathological analysis, although with relatively low direct osseointegration of the implant. In conclusion, dexamethasone does not increase the risk of developing periprosthetic osteolysis or infection in a pre-clinical model of ODRI. Long-term administration of dexamethasone seemed to offer a benefit in terms of new bone formation around the implant, but with low osseointegration.

## 1. Introduction

Total joint arthroplasties are among the most common surgical procedures performed worldwide. In 2015, over 1 million total hip (THA) and knee (TKA) prosthesis were implanted in the United States [1], and numbers are expected to increase in future years. These operations are highly standardized and have a high success rate and patient satisfaction. In some clinics, outpatient THA and TKA programs are being developed for selected patient groups, with promising initial results [2,3]. Two of the many limitations to successful outpatient joint replacement are postoperative pain and postoperative nausea and vomiting (PONV) [4]. The use of preoperative low-dose dexamethasone, a synthetic glucocorticoid, has been shown to decrease the incidence of PONV and reduce hospital length of stay (LOS) of patients undergoing arthroplasty [5,6] and is therefore commonly used in this setting.

Glucocorticoids are a class of cholesterol-derived hormones physiologically produced in the adrenal cortex of mammals. Cortisol, the principal glucocorticoid in humans, is of utmost relevance for the systemic regulation of several complex systems, maintaining, among other things, pro- and anti-inflammatory mediators in proper balance [7] such as restoring homeostasis after acute inflammation. Synthetic glucocorticoids are a class of drugs which were first used clinically in the 1950’s for substitution in the case of adrenal insufficiency. Synthetic glucocorticoids have been shown to reduce proinflammatory cytokines and to activate anti-inflammatory cytokines, thus reducing the inflammatory response [8]. Synthetic glucocorticoids are currently widely used as anti-inflammatory agents in the treatment of a wide array of chronic inflammatory diseases such as rheumatoid arthritis, inflammatory bowel disease, or chronic asthma [9]. For those conditions, a chronic treatment of glucocorticoid is typically administered (e.g., 40–60 mg oral prednisolone/day for Crohn’s disease, with a gradual dose reduction over time). It is estimated that 0.5–1% of the general population is chronically treated with synthetic glucocorticoids [10].

Theoretical concerns about safety of use of synthetic glucocorticoids preoperatively, especially in relation to potential increased postoperative infection rates, do not seem to be supported by the clinical literature [11,12]. The clinical evidence regarding a potential correlation between chronic use of synthetic glucocorticoids and an increased rate of periprosthetic joint infection (PJI) are inconclusive [13,14]. It has been shown, for example, that patients on chronic glucocorticoid treatment have an increased risk of revisions at 90 days after implantation of a THA, regardless of the indication for the revision (i.e., infection or aseptic complication) [15]. Furthermore, it has been established that chronic glucocorticoid treatment increases the risk of developing osteoporosis [16], but it has not been established whether the osseointegration of orthopedic implants or the risk of prosthetic loosening is different in these patients than in the general population.

Since the clinical literature is not available to address these questions, pre-clinical assessment of a potential impact of steroid administration on implant osseointegration or infection rate in orthopedic surgery would be a welcome addition. In this study, an experiment was performed to establish an acute postoperative Orthopedic-Device-Related Infection (ODRI) in an animal model, with the aim of analyzing the possible impact of the administration of dexamethasone, in a first phase after a single preoperative administration to determine whether the risk of developing an infection is changed and in a second phase in the context of daily dexamethasone administration to see if chronic administration interferes with the treatment of infection or osseointegration.

## 2. Materials and Methods

### 2.1. Study Outline

Consent to perform the study was granted by the ethical committee of the canton of Graubünden, Switzerland (approval number 04/2019), and was performed in an AAALAC-accredited research institute. The model was based on a previously published study of ODRI [17,18,19], where a polyether ether ketone (PEEK) screw contaminated with *S. epidermidis* was inserted in the proximal tibia of rats. The interface between the bone and the implant was monitored with transversal in vivo microCT-scanning (μCT) and bacteriology after euthanasia. A total of 44 animals were included. PEEK screws, either sterile or contaminated with *S. epidermidis*, were implanted into the left tibia, as described below, at 22 ± 3 weeks of age.

In the first experiment, designated as the risk study, the model was used to assess the potential impact of a pre-operative single-shot of synthetic glucocorticoid on the likelihood of developing ODRI after implantation of a contaminated screw. A total of 20 animals were used for this phase. Each animal received a PEEK screw contaminated with 1.2 × 10^2^ CFU of *S. epidermidis*, implanted in the proximal tibia. This inoculum was selected to be at the threshold for ability to induce an infection an so easier to determine if infection risk was increased or decreased after treatment compared with controls. The animals were divided into two groups, each consisting of 10 individuals. The first group received a single shot of subcutaneous dexamethasone (0.3 mg/kg BW) 30 min prior to the surgical procedure, while the other group denoted as the control (CTL) group received a placebo injection of saline. This dose of dexamethasone was selected on the basis of human clinical medicine where a pre-operative dose of more than 0.2 mg/kg BW significantly reduces pain on mobilization. The duration of the experiment was 9 days. MicroCT-Scanning was conducted on days 0 and 9. Quantitative bacteriology of the bone, soft tissue and implant was conducted after euthanasia.

In the second experiment, designated as the interference study, the model was used to investigate the potential influence of chronic administration of synthetic glucocorticoid on the outcome of antibiotic treatment of an established ODRI. This part of the study was conducted for 28 days and involved 24 animals. Each animal received a PEEK screw contaminated with 1.5 × 10^6^ CFU of *S. epidermidis* surgically implanted in their proximal right tibia. The higher inoculum was selected to ensure an ODRI was established, so the effect on treatment outcome could be observed. Among these animals, the first group of 12 animals received a daily subcutaneous injection of dexamethasone (0.3 mg/kg BW), while the second group (CTL) received a daily subcutaneous injection of saline. All animals were treated with antibiotics from day 7 to day 21 (rifampin (25 mg/kg BW s.c. 2×/day) and cefazolin (30 mg/kg BW s.c. 2×/day). The strain of *S. epidermidis* examined as part of our research is sensitive to this antibiotic, and treatment in the same model has previously been found to be effective [19]. Furthermore, both antibiotic agents are routinely used for treatment of bone infection, particularly for sensitive strains of *S. epidermidis*. The assessment of osteolysis, implant osseointegration, and periosteal reaction was conducted through serial μCT-scanning at days 0, 9, 20, 28; quantitative bacteriology of the bone, soft tissue and implant was conducted after euthanasia. Six randomly chosen animals (3 from each group) were allocated for semi-quantitative histopathological as well as quantitative histomorphometric analysis and thus were excluded from quantitative microbiological assessments. Table 1 gives an overview of the entire study.

### 2.2. Implant Design

The implants were fabricated using medical grade PEEK by RISystem AG, Davos, Switzerland. The implants were 5 mm in length and 1.5 mm in diameter. To facilitate visualization within the microCT-scanner, the PEEK had 20% (by weight) barium sulfate, a contrast agent supplied by Invibio Biomaterials Ltd. (Thornton-Cleveleys, UK).

### 2.3. Bacterial Inoculum Preparation

The bacterium used for the experiment was *S. epidermidis* strain 103.1. This strain is a clinical isolate from a patient with a chronic ODRI, available from the Culture Collection of Switzerland, strain number CCOS 1152. For each experiment, the isolate was recovered form frozen stocks and cultured on tryptic soy agar (TSA, Oxoid, Basel, Switzerland) at 37 °C. The screws were contaminated 30 min prior to each surgery by submerging the threaded portion for 25 min at room temperature in a suspension prepared as follows: overnight cultures of *S. epidermidis* were centrifuged (2500 g for 10 min), the pellet was washed twice in phosphate-buffered saline (PBS) and adjusted to an optical density of 0.50 (±0.01) at 600 nm. A series of serial dilutions in PBS was performed to achieve the desired concentration of CFU to contaminate the screws at the desired level, according to the study protocol. Parallel to each series of inoculation, test screws were inoculated for controlling the *S. epidermidis* adhesion. These test screws were sonicated in PBS for 3 min and subsequently plated on 5% horse blood agar (Oxoid) after serial dilution. The inoculum was quantified after overnight incubation at 37 °C.

### 2.4. Animal Welfare, Observation, and Euthanasia

Skeletally mature, female, Wistar, specific-pathogen-free rats (mean age 22 ± 3 weeks with an average weight of 316.3 g ± 20.4 g (risk study, Control group), 312.6 g ± 28.4 (risk study, Dexamethasone group), 327.7 ± 20.7 g (interference study, Control group), and 301.7 ± 11.4 g (interference study, dexamethasone group) were used. The rats were purchased from Charles River, Cologne, Germany. All animals were group-housed in ventilated cages with a 12 h light/dark cycle (3–4 animals per cage). The animals had access to water and food ad libidum (Granovit AG, Kaiseraugst, Switzerland). To monitor animal welfare postoperatively, the rats were scored by an animal care giver or veterinarian twice a day for 5 days followed by once a day until day 7. Further scoring was performed twice a week for the remainder of the study. Parameters which were evaluated included behavior, breathing, external appearance, weight bearing of the operated leg, excretion, wound healing, and bodyweight.

The dexamethasone dose and antibiotics were given on a weight adjusted basis. Weights were taken twice a week, and dosage adjusted per the most recent weight at all times. Animals were euthanized after 9 days for the risk study or 28 days for the interference study. Euthanasia was performed by intracardiac injection of a lethal dose of pentobarbital under sevoflurane anesthesia.

### 2.5. Surgery

Induction of anesthesia was performed by transferring the rats in an induction box exposing them to 8% sevoflurane in 100% oxygen. Anesthesia was maintained with 2.5% sevoflurane in 100% oxygen using a face mask. Oxygen flow was set at approximately 700 mL/minute. For analgesia, the rats received buprenorphine (0.03 mg/kg) subcutaneously prior to surgery.

Preoperatively, the right leg was clipped, and the rat was placed in the supine position on a heating pad. The right leg was aseptically prepared. After a skin incision on the proximal lateral aspect of the tibia, the patellar ligament and growth plate were identified. A unicortical hole was drilled by hand 2 mm distal to the growth plate using a custom-made stop guide (PEEK Screw Drill Stop Sleeve 3 mm; RIS.390.360-05). The contaminated screw was then inserted. The fascia and skin were closed in two layers. Monocryl sutures (6-0, Ethicon, Somerville, NJ, USA) were used for fascia using a continuous pattern. In total, 5-0 Vicryl rapide (Ethicon) was used for the skin using an intracutaneous suture technique.

For postoperative analgesia, paracetamol (Dafalgan Kindersirup 30 mg/mL, Bristol-Meyers Squibb, Princeton, NJ, USA)was added to the water dispenser for the first 5 days postoperatively (7 mL Dafalgan sirup/100 mL water).

### 2.6. In Vivo MicroCT and Image Processing

The bone, implant osseointegration and periosteal reaction were monitored using serial MicroCT-scanning immediately after surgery and at euthanasia using methods previously described [18]. Additionally, during the interference study, micro-CT scanning was conducted on days 9 and 20. The scanning was performed under isoflurane anesthesia. A 10 mm region of interest (ROI) centered on the implant was scanned at a resolution of 25 µm. The image processing was performed to determine bone resorption (BR), new bone formation (BF), bone fraction (Bone Volume/Total Volume, BV/TV) as well as a bone–implant contact (BIC) rate in a ROI of 700 µm around the surface of the threaded part of the implant. The periosteal new-bone formation was assessed 2 mm proximal and distal from the screw head, i.e., outside of the above defined ROI. Image processing and analysis were performed with EasyIPL (www.easyipl.com, accessed on 1 July 2020), a library of macros using the scanner software (SCANCO image processing Language, IPS and OpenVMS Digital Command Language, DCL, version 9.2).

### 2.7. Bacteriology

After euthanasia, the tibiae were dissected, and the fibrous soft tissue overlying the screw head, the screw itself and the bone were isolated in separate sterile containers filled with sterile PBS. The implant was sonicated for 3 min and vortex-mixed for 10 s in PBS. The entire bone as well as the soft tissue were mechanically homogenized (Omni Tissue Homogenizer and Hard Tissue Homogenizing tips, Omni International, Kennesaw, GA, USA). All samples were then subjected to serial dilution and plated on blood agar. The agar plates were incubated for 24 h at 37 °C and checked after 24 h for signs of contamination.

### 2.8. Histological Processing

Following euthanasia, six animals from the interference study (three from the control group, three from the dexamethasone group) were randomly chosen and assigned to histological analysis. After removal of the overlying skin, the lower limb was fixed for a minimum of two weeks in 70% methanol. Following this, dehydration was completed with serial ascending ethanol series (70%, 96%, absolute ethanol), with two changes for each step every 2–5 days. The specimens were then transposed to xylene and were finally embedded with methylmethacrylate (MMA; Sigma Aldrich, Buchs, Switzerland). The samples were sectioned with an annular blade saw (Leica 1600, Leica Biosystems, Nussloch, Germany). A minimum of two sections were made in the frontal plane through the center of the implant, with one section stained with Giemsa-Eosin and one section with Brown and Brenn to detect Gram-positive bacteria.

### 2.9. Histopathologic Analysis (Semi-Quantitative)

The samples were assessed by a certified veterinary pathologist (DN). A semi-quantitative method (grade 0–5) was used focusing on parameters for infection, inflammation, bone formation, and implant integration. No statistical analysis was performed due to the limited specimen number (n = 3). This assessment was completed to give a general morphologic overview of the compared treatments.

### 2.10. Histomorphometric Analysis (Quantitative)

**Figure 1:**
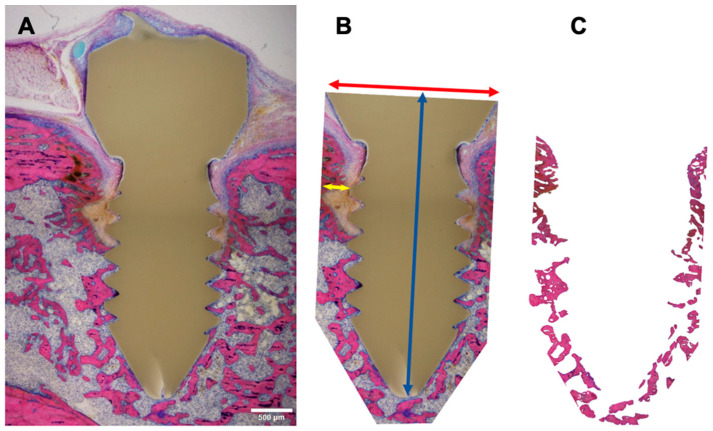
Histomorphometric measurements. (**A**) Reference sample: overview image of an animal from control group. (**B**) Region of interest, ROI: Non-serrated sleeve around the implant with approximately 300 µm to the tips of the threads (yellow arrow). Its width (red arrow) is defined by the width of the screw head at its neck (approx. 2090 µm) and its height (blue arrow) by the length of the screw from the neck to the tapered tip (approx. 3443 µm). (**C**) Bone marking: bone area inside the ROI marked interactively by Photoshop’s “magic wand” tool. MMA-embedded, GE-stained thick-sections; objective: 4× (stitched); scale bar 500 µm, calibration 0.452 pixels/µm.

The amount of bone adjacent to the implant was analyzed with a quantitative histomorphometric method (Figure 1). On each digital image, the bone area inside a sleeve of 300 µm around the implant was measured using Adobe Photoshop 2020 (including analysis plug-in), with a calibration of 0.452 pixels/µm. The region of interest (ROI) was defined by using the image of the first control sample animal (the identical ROI was applied for the analysis of all other samples) and resulted in a polygon around the implant with an area of approximately 6.6 mm^2^ (exact 6,654,197 µm^2^). Its width was defined by the maximum width of the screw head at its neck (approx. 2090 µm) and its height by the length of the screw from the neck to the tapered tip (approx. 3443 µm). The bone area inside the ROI was marked by the Photoshop tool interactively to avoid marking non-bone areas with similar grey values (settings: point sample, tolerance 50, continuous area, grey value min [mean]: 21.1, grey value max [mean]: 154.4).

### 2.11. Statistical Analysis

Quantitative bacteriology data were analyzed using unpaired *t*-tests. Differences in proportions between groups were analyzed using the Fisher Exact Test. MicroCT bone parameters over time were assessed as mean ± SEM and analyzed using for each time point with a paired *t*-test. Statistical significance threshold was set as *p* < 0.05. Statistical analyses were completed with GraphPad Prism software Version 9.4.0 (453) (GraphPad Software, Inc., La Jolla, CA, USA).

## 3. Results

### 3.1. Animal Welfare

All animals recovered well from surgery and anesthesia. Mean weight loss at 3 days following surgery was 5.7% in the treatment group and 4.0% in the control group of the risk study and 2.8% in the treatment group and 2.2% in the control group of the interference study.

### 3.2. Effect of Dexamethasone on Bacterial Burden

Quantitative bacteriology was performed at the end of both studies to quantify the viable bacteria present in the proximity of the implanted PEEK screw. In the risk study, one in ten rats was culture-negative in the control group, whereas all animals showed bacterial growth in the dexamethasone-treated group. There was no statistically significant difference between the mean CFU counts in the group treated with dexamethasone compared to the control group (mean CFU count ± SEM: dexamethasone = 3590 ± 3350 CFUs; control = 3030 ± 2491 CFUs, *p* = 0.894).

**Figure 2:**
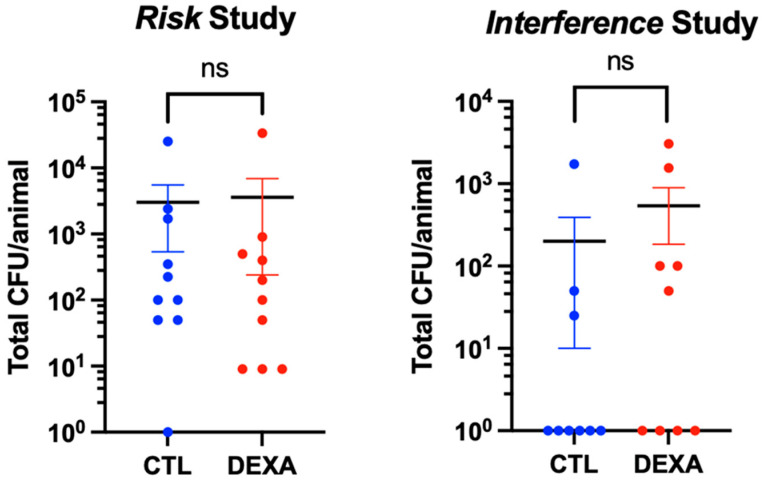
Total bacterial load per animal at euthanasia, i.e., 9 days after surgery, risk study. Data shown are the mean ± SEM. Unpaired *t*-tests were performed to assess significant differences between dexamethasone-treated and control groups.

In the interference study, six out of nine rats were culture-negative in the control group, while four out of nine were culture-negative in the dexamethasone group. There was no statistically significant difference between the mean CFU counts in the group treated with dexamethasone compared to the control group (mean CFU count ± SEM: dexamethasone = 539.3 ± 355.7 CFUs; control = 200.7 ± 190.6 CFUs, *p* = 0.414) (Figure 2).

### 3.3. Cancellous Bone Changes in Response to Preoperative Dexamethasone Administration

**Figure 3:**
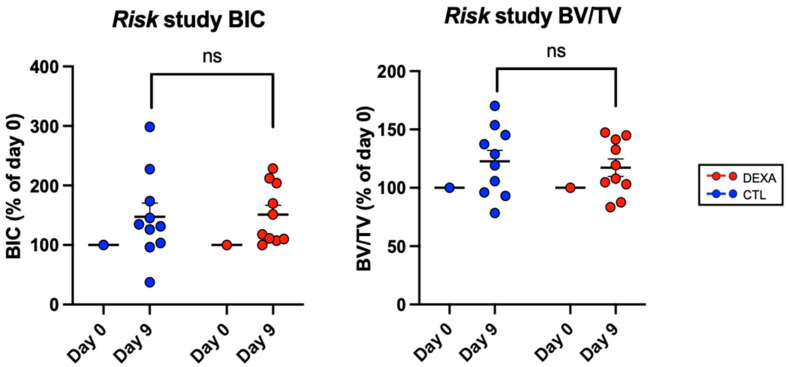
Bone changes in response to dexamethasone therapy at surgery and at euthanasia, i.e., 9 days after surgery, risk study. Data shown are the mean ± SEM. Paired *t*-tests were performed to assess significant differences between dexamethasone-treated and control groups.

In the risk study, we evaluated whether preoperative administration of a dose of dexamethasone would have an impact on the initial osseointegration of an implant in cancellous bone, as well as possible perifocal osteolysis. MicroCT analysis showed no significant difference in BIC or BV/TV at 9 days after surgery (Figure 3).

### 3.4. Cancellous Bone Changes in Response to Antibiotic Therapy and Continuous Dexamethasone Therapy

**Figure 4:**
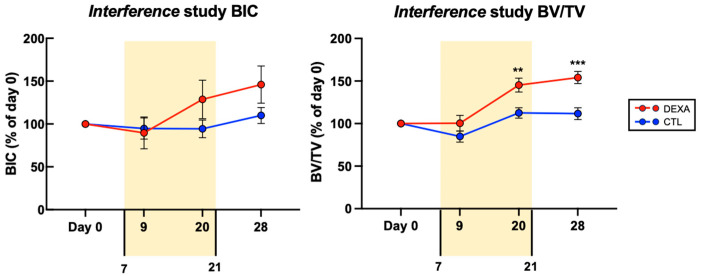
Bone changes in response to antibiotic and dexamethasone therapy over time, interference study. The period during which the antibiotics were administered, i.e., starting at day 7 and discontinued at day 21, is shown in yellow. Data shown are the mean ± SEM. Paired *t*-tests were performed to assess significant differences between dexamethasone-treated and control groups for each time point. ** *p* < 0.01, *** *p* < 0.001.

**Figure 5:**
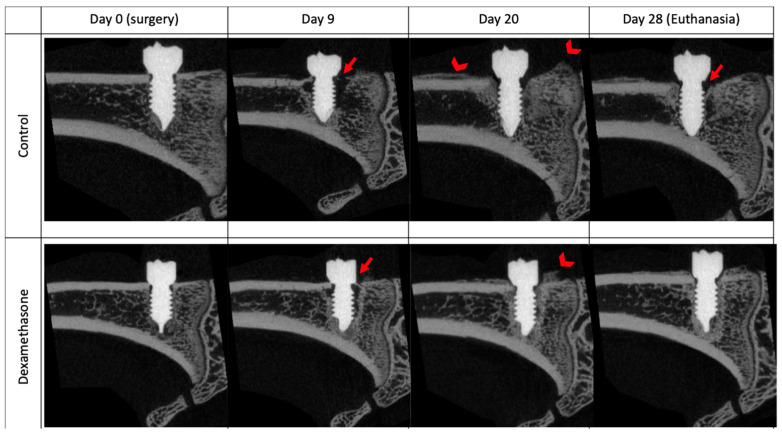
Illustrative time series of in vivo microCT scans from the interference study. The upper line shows the scans of an animal from the control group. The lower line shows the scans of an animal treated with 0.3 mg/kg BW dexamethasone daily. The lines of images are each taken from the same animal. The depicted scans were chosen based on median BV/TV values from each experimental group. Arrows indicate regions of osteolysis, arrowheads show periosteal reaction.

In the interference study, we evaluated the impact of a combined treatment comprising dexamethasone and antibiotics on the bone changes during *S. epidermidis* infection. The inclusion of dexamethasone did not result in a significant alteration in *S. epidermidis*-induced changes in BIC. However, we noted a statistically significant higher bone fraction (BV/TV) at day 20 and day 28 in the dexamethasone-treated group than in the control group. The magnitude of the difference is particularly marked during the phase in which the antibiotic treatment was administered and seems to remain stable once the antibiotic is discontinued. (Figure 4 and Figure 5).

### 3.5. Histomorphometric Analysis

**Figure 6:**
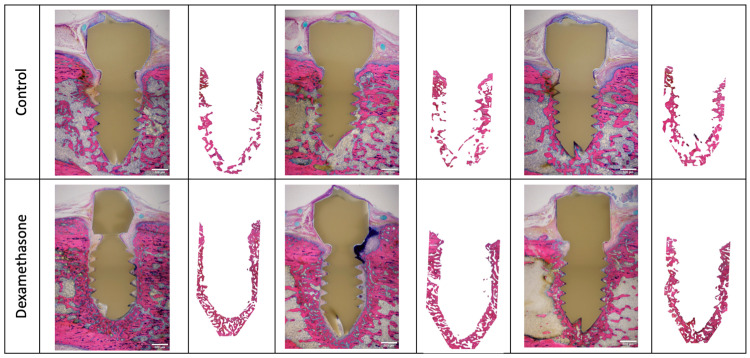
Histomorphometric assessment of bone changes from *S. epidermidis*-contaminated screws in the absence or presence of dexamethasone treatment after 28 days. Scale bars 500µm.

Histomorphometric analysis was conducted in three randomly chosen animals from each group in the interference study. Dexamethasone administration increased the amount of bone tissue present near the implant by 31.5% compared to control animals (mean bone area ± SD: control = 1.098 ± 0.166 mm^2^; dexamethasone = 1.444 ± 0.144 mm^2^) (Figure 6).

### 3.6. Histopathologic Analysis

None of the animals, whether in the control group or the dexamethasone-treated group, displayed evidence of multifocal or diffuse inflammation of the bone marrow (myelitis with/without micro abscess formation). The bone marrow exhibited a diffuse, low-grade, activation, predominantly myelopoietic, in 3/3 animals in the control group, and 2/3 of animals in the dexamethasone group.

Remarkably, 2/3 animals treated with dexamethasone demonstrated minor necrotic changes of small, fragmented/sequestered bone regions near the implant. In both of these animals, there is a sleeve measuring around 40 µm between the bone and the implant, filled with inflammatory and necrotic material. It is striking to note that the bone does not penetrate the space between the individual threads of the screw in these two animals. None of the control group animals showed any indications of bone necrosis. All the animals with evidence of necrosis (2/3 animals) had Giemsa-positive coccoid bacteria detected in the Giemsa–Eosin-stained sample.

## 4. Discussion

Joint arthroplasties have become some of the most frequently performed surgical procedures worldwide. These interventions aim to alleviate pain, enhance mobility, and improve the quality of life for individuals with debilitating joint conditions. Despite the success of joint arthroplasties, they are not without challenges. Complications, such as infection, implant failure, and the need for revision surgeries, still occur and are associated with a significant physical and mental burden for the patient and a significant financial burden for healthcare systems [20,21].

This pre-clinical study focused on two distinct scenarios of relevance to all orthopedic devices. In the first, we simulated the administration of a single shot of dexamethasone in the preoperative period before implantation of a screw contaminated with a low count of *S. epidermidis* in the tibia of rats. This administration did not change the incidence or magnitude of infection in the treated group compared to the control group. There was also no impact on osseointegration or bone response around the implant at 9 days measured by microCT. This research question was based on human clinical studies [5,6] showing that the administration of dexamethasone prior to joint replacement reduces postoperative pain and postoperative nausea and vomiting (PONV). Pain and PONV are notable limitations in the context of joint replacement procedures, especially in an outpatient setting. PONV is a complication causing a decrease in patient satisfaction, delaying mobilization after arthroplasty, and ultimately increasing LOS [22]. Given the effectiveness of preoperative administration of dexamethasone in the prophylaxis of PONV in joint replacement surgery, several guidelines, such as the “Consensus statement for perioperative care in total hip replacement and total knee replacement surgery” from the Enhanced Recovery After Surgery (ERAS) society, recommends its systematic use [23]. Despite these advantages, surgeons remain concerned about the potential impact of dexamethasone administration on the risk of developing an infection after arthroplasty. Given the catastrophic consequences of PJI for the patient and the cost to the healthcare system, it seems of the utmost importance to ensure maximum patient safety and to carefully study the modifiable aspects that would tend to reduce the probability of its occurrence.

Even if no pre-clinical models have been studying the impact of a single shot of dexamethasone preoperatively on the risk of developing ODRI, the literature provides clinical studies based on arthroplasties dealing with this issue. None show a trend towards increased infection in the groups that received a single shot of dexamethasone. Four of these studies have important findings. In 2021, 91 total shoulder arthroplasties were analyzed in a randomized, controlled trial. The patients of the treatment group (n = 43) received 10 mg i.v. dexamethasone 90 min before surgery and were compared with a control group of 32 patients. The study showed a significantly better pain control, with significantly lower opioid consumption in the first 24 postoperative hours in the treatment group. The average LOS was not significantly different. There was no difference in the postoperative infection rate at 3 or 18 months [24]. However, the main outcome analyzed was not the development of PJI, and the study was therefore not powered sufficiently to be conclusive. In 2019, a retrospective analysis of 856 THA or TKA, in which 57.5% were preoperatively treated with dexamethasone, showed a significant reduction in PONV in the dexamethasone group, with an associated diminution in the LOS. There were no differences in the postoperative infection rate at 30 or 90 days [5]. In 2018, a retrospective analysis of 7910 THA/TKA (1293 dexamethasone group, 6617 control group) showed no difference in the PJI risk at 90 days postoperatively [1]. Finally, a large retrospective analysis of more than 18,000 THA and TKA by Vuorinen et al. in 2019 and focusing specifically on the infection safety of preoperative administration of dexamethasone came to the conclusion that this practice has no impact on the risk of developing an PJI [25]. It is worth noting the average follow-up of 4.9 years in this study. Our pre-clinical study seems to confirm this conclusion in an animal model of contaminated screw implantation.

In a second scenario, we established an ODRI using an implant highly contaminated with *S. epidermidis*, then treated this early ODRI with antibiotics only, and compared a group of control animals to a group treated with dexamethasone daily. This reflects a clinical situation whereby a patient chronically treated with glucocorticoids receives an orthopedic implant. These may typically be patients treated for rheumatoid arthritis (RA), a group of patients who benefit from reduced joint pain and improved joint function through arthroplasty but are prone to significantly more PJIs than patients unaffected by this disease [26]. While it cannot be established whether RA or its treatment increases the risk of infections, it does seem that patients chronically treated with glucocorticoids have an increased risk of PJI [15,27]. Our controlled, preclinical study shows no statistical difference in terms of magnitude or incidence of infection in the two groups in this scenario measured by bacteriology. MicroCT imaging revealed that the Bone Implant Contact (BIC), which can be interpreted as a marker of implant osseointegration, does not show a significant difference between the two groups throughout the 28 days experiment. Meanwhile, the bone fraction (BV/TV) around the implant was significantly greater in the dexamethasone group than in the control group.

However, the analysis of these markers of bone presence in the microCT-scan must be carefully moderated and correlated with the analysis of the histological images. Even if a higher amount of bone area was observed in the dexamethasone-treated group compared to the control group 28 days after surgery, a closer look reveals larger areas of soft tissue around the threaded part of the implant in two of the three images available. In those animals, the bone does not grow into the space between the individual threads, and there is no direct contact to the implant. This finding is not consistent with the BIC analysis of the microCTs and highlights the limits of microCT as a reliable marker of osseointegration. This large degree of indirect (= fibrous) integration is of concern, as it could indicate that in the case of ODRI developing in the early postoperative period in a patient chronically treated with dexamethasone, there could be a significant risk of early loosening of the implant. Given that it has been suggested that FRI [28] or PJI [29] with a “short” infection time after index surgical intervention (typically less than 28 days postoperatively) could be treated by debridement, antibiotics and implant retention (DAIR), our study seems to suggest that this approach might be more risky for patients chronically treated with glucocorticoids and that the indication for implant removal might be given more aggressively.

The histopathological images are striking for the sleeve at the interface between the implant and the cancellous bone, but also for the density of the cancellous bone around the implant after 28 days, in particular since it has been established that chronic treatment with glucocorticoids tends to cause the opposite, i.e., a loss in bone density (clinically known as osteoporosis) [30]. This has also been shown in pre-clinical studies in which dexamethasone was administered to rats without the addition of infected intraosseous material, where substantial loss of cancellous bone volume after 3 months of administration was observed [31].

It seems to us that two factors could explain this paradoxical phenomenon: Firstly, the hypothetical existence of a biphasic effect of dexamethasone on the mitochondrial metabolism of osteoblasts and osteoclasts at the start of treatment. In our study, the animals were naive to dexamethasone and only began to receive it at the start of the experiment. This is a fundamental difference from patients treated chronically with glucocorticoids, in whom one would implant a joint prosthesis or osteosynthesis material after years of exposition to glucocorticoids. It is possible that dexamethasone-naive osteoclasts reduce their capacity to resorb bone substances in the first 24 h after first contact with dexamethasone, as it has been shown in vitro [32]. This effect could be due to the reduction in mitochondrial activity observed in osteoclasts in the first 24 h after dexamethasone administration during an experiment carried out in vitro by a group of Taiwanese researchers [33]. In a following paper, this same group demonstrated a biphasic effect of dexamethasone on the activity of osteoblasts, with an increase in their mitochondrial activity during the first 24 h after administration [34]. This cumulative effect of decreased resorption and increased bone production, which could have occurred during the first few hours after screw implantation, could explain the increased cancellous bone density in our histopathological images. It should be noted that the difference in BV/TV between the treated group and the control group in our microCT analyses only became significant 20 days after the start of the experiment, and the difference increased thereafter. This may corroborate this theory of a paradoxical effect of dexamethasone exposure insofar as the time taken for osteoclasts to resorb a portion of injured bone takes about 3 weeks, whereas the formation of new bone takes about 2–3 months [35] (i.e., the lack of resorption in dexamethasone-treated animals becomes apparent after three weeks, compared with animals in the control group, while at the same time, the increased activity of osteoblasts from the start of the experiment also becomes evident after three weeks, compared with the ‘non-super-activated’ osteoblasts in the control group).

Second, the anti-inflammatory activity of the glucocorticoid leads to the retention of debris in the vicinity of the implant. In two out of three animals in the dexamethasone-treated group, necrotic bone fragments were observed sequestered in the vicinity of the implant in the histopathological images. This retention of debris was not observed in any of the animals in the control group. Necrotic bone debris are physiologically phagocytosed by osteoclasts, macrophages and neutrophils [36]. In the previous paragraph, we discussed the potential role of dexamethasone at the beginning of its exposition in reducing the capacity of osteoclasts to resorb bone substances. Further, it has been shown that glucocorticoids in general and dexamethasone in particular lead to apoptosis of monocytes and prevent their differentiation into tissue macrophages [37]. It is possible that, as a result of this insufficient clearance of debris in the vicinity of the implant, the remains of necrotic tissue have prevented the advance of bone formation in the immediate vicinity of the implant, leading to the formation of the sleeve observed on histology and preventing osseointegration.

Consequently, we postulate that on the one hand, in the immediate vicinity of the implant where the presence of bacteria was greatest, dexamethasone prevented osteoclasts, macrophages and neutrophils from combating the bacteria and clearing away the necrotic bone particles present as a result of the surgical manipulations. This led to the formation of the sleeve between the cancellous bone and the implant. On the other hand, the effects of primary exposure to dexamethasone outside the directly contaminated area led to the development of much denser cancellous bone through osteoblastic hyperactivity and osteoclastic hypoactivity.

### Limitations

This study uses PEEK implants, and the osseointegration reaction at the interface with materials used in orthopedic surgery (e.g., cobalt-chromium, titanium) may be different. Furthermore, in the interference study, infections were treated with antibiotics only. This differs from current clinical recommendations. Current accepted practice for the treatment of ODRI is either the replacement/removal of infected material, or DAIR, which consists of the administration of antibiotics in conjunction with surgical debridement and, if applicable, replacement of moving parts. In addition, the use of parenteral corticosteroids as used here may be less common than the oral route. The parenteral route was selected in this study due to animal handling reasons; although, this would be a valid future comparison study. Finally, our histopathological analysis is based on only three animals chosen at random from each group, which does not allow for rigorous or statistical analysis.

The results do show, nevertheless, a potentially positive effect of dexamethasone therapy on bone metabolism in vivo. Further research that would be relevant include a dose-ranging study to monitor the effect at a range of doses. It would also be interesting to carry out the same experiment implanting non-infected screws and analyzing all the animals by histopathology, in order to observe whether the increase in bone density observed in this study also leads to increased osseointegration in the case of non-contaminated material implantation. If the results are conclusive, a clinical study could then be designed on osteosynthesis with a low risk of infection, such as osteosynthesis for closed fractures of the distal radius or closed fractures of the proximal femur, to study whether a short course of dexamethasone therapy leads to accelerated bone healing.

## 5. Conclusions

This pre-clinical study leads to two important conclusions. Firstly, it provides further evidence that pre-operative single-shot administration of dexamethasone prior to orthopedic device implantation has no influence on the risk of infection development or on the early phases of osseointegration of the device. Secondly, there is an effect on the direct contact zone between an implant and cancellous bone in the event of infection and concomitant chronic administration of dexamethasone. This point is of concern, and we believe it is necessary to evaluate it through clinical studies, in particular to assess the failure rate of DAIR treatment in short-term (e.g., <28 days after index surgery) infection in patients treated chronically with glucocorticoids, whether in the context of PJI or FRI.

## Figures and Tables

**Table 1:** Overview of study design.

Study Groups	Duration	Inoculum	Treatment	Antibiotics	Rats (*n*)
**Phase 1, Risk**					**20**
DEXA	9 days	1.2 × 10^2^ (CFU/Screw)	Dexamethasone 0.3 mg/kg BW, once preoperatively	None	10
CTL			Saline 0.1 mL, once preoperatively		10
**Phase 2, Interference**					**24**
DEXA	28 days	1.5 × 10^6^ (CFU/Screw)	Dexamethasone 0.3 mg/kg BW, daily	Rifampin and Cefazolin	12
CTL			Saline 0.1 mL, daily		12
**Total**					**44**

## Data Availability

The data presented in this study are available on request from the corresponding author.
